# Ion-Doped Silicate Bioceramic Coating of Ti-Based Implant

**DOI:** 10.7508/ibj.2016.04.002

**Published:** 2016

**Authors:** Hossein Mohammadi, Mohammadmajid Sepantafar

**Affiliations:** 1 School of Materials and Mineral Resources Engineering, Universiti Sains Malaysia, Engineering Campus, 14300 Nibong Tebal, Penang, Malaysia; 2 Department of Stem Cell and Developmental Biology, Cell Science Research Center, Royan Institute for Stem Cell Biology and Technology, ACECR, Tehran, Iran; 3 Department of Metallurgy and Materials Engineering, Faculty of Engineering, University of Semnan, Semnan, Iran

**Keywords:** Plasma spray, Modified silicate ceramics, Coating, Ti implant

## Abstract

Titanium and its alloy are known as important load-bearing biomaterials. The major drawbacks of these metals are fibrous formation and low corrosion rate after implantation. The surface modification of biomedical implants through various methods such as plasma spray improves their osseointegration and clinical lifetime. Different materials have been already used as coatings on biomedical implant, including calcium phosphates and bioglass. However, these materials have been reported to have limited clinical success. The excellent bioactivity of calcium silicate (Ca-Si) has been also regarded as coating material. However, their high degradation rate and low mechanical strength limit their further coating application. Trace element modification of (Ca-Si) bioceramics is a promising method, which improves their mechanical strength and chemical stability. In this review, the potential of trace element-modified silicate coatings on better bone formation of titanium implant is investigated.

## INTRODUCTION

One of the most successful economical and surgical procedures for bone tissue repair is total joint replacement. This procedure could enhance function and movement and decrease pain in patients suffering from severe arthritis and skeletal tissue abnormalities[1].

The successful performance of biomedical implant mainly relies on the suitable osseointegration at the interface of host tissue and biomaterial[2]. Osseointegration is occurred when functional integrity is created directly between the bone tissue and the surface under load implant[3].

Ti-6Al-4V is a well-recognized biomaterial with proper mechanical features and biocompatibility, which are found in many biomedical implants such as bone screw. However, the lack of biodegradability, the slow rates of osseointegration and poor mechanical anchorage result in implant failure and loosening[4-9]. Furthermore, a fibrous layer is formed at the interface between the implant and tissue. Also, local inflammation and infection are occurred most probably due to the long-term presence of implant *in vivo*[10].

The available synthetic implants still have restrictions in clinical practice and need revision surgery due to the formation of thick fibrous tissue at the tissue-biomaterial interface[11,12]. The revision surgery decreases the quality of the life of people suffering from hard tissue diseases, since it is more difficult than the initial surgery. Many attempts have been performed on the quality of available biomedical implants by surface modification. As stated above, development of new implants coated with bioactive and functionally stable materials is necessary. Different surface modification methods have been employed to modify the surface of currently available biomedical metallic implants[13]. The coating materials play an important role in providing an environment in which bone formation ability is enhanced and in turn, better integration is established between the implant and bone tissue. Various surface modification methods have been used to encourage the bone formation between tissue and medical implant[14-16], including chemical vapor deposition[17,18], anodic oxidation[19], sol-gel[6,20], physical vapor deposition[6,21], plasma spray[22], electrophoretic deposition (EPD)[23], anodic spark deposition[2] and enameling[24,25].

Bioceramics, such as calcium phosphate[26], hydroxyapatite (HA)[27,28] and calcium silicate (Ca-Si)[29] have been used as coating materials on the surface of biomedical implants. HA could directly bond with the bone tissue with no fibrous layer formation[27,28]. However, it possesses low osteogenic activity[30,31], inadequate chemical stability[32,33], mismatch of thermal expansion coefficient (CTE) with Ti-6Al-4V substrate[34,35] and low bonding strength[36,37], which lead to short-term osseointegration. The mismatch of CTE between HA coating and Ti substrate provides higher tensile strength at the interface, decreases the bonding strength of coating and may cause peeling and fatigue failure under tensile loading[38]. Also, bioglasses[39,40] have been applied to modify the surface of medical implants. However, most of the bioglasses coatings have poor bonding strength due to the mismatch of their CTE with Ti-6Al-4V[41] and high degradation rate[42].

Ca-Si-based ceramics have shown to have higher bonding strength with Ti substrate compared to HA[29]. Further, they could support osteoblast attachment as well as proliferation and differentiation by the release of calcium (Ca^2+^) and silicon (Si^2+^) ions[43-45]. Also, the dose-dependent antibacterial activity of Ca-Si-based ceramics has been also demonstrated in some studies[46,47]. Silicate bioceramics possess comparable CTE with Ti-6Al-4V; as a result, the high bonding strength is provided and also the residual stress is decreased[35,48,49]. However, their chemical instability, inability to support human bone formation and poor mechanical properties limit their applications as a biomedical coating for long-term orthopedic implants[50].

It has been reported that the positive ion modification (trace element) improves the biological and mechanical strength of Ca-Si-based ceramics[51,52], which may increase their bone bonding ability[53,54]. Therefore, it is reasonable to use trace element-incorporated silicate bioceramic as coating materials for metallic implants. The objective of this review is to investigate whether the ion-modified Ca-Si coating can effectively improve the osseointegration of implant and, in turn, the quality of life of patients compared to conventional ceramic coatings.

### Various characteristics of ideal biomedical coating Structural properties

A coating material with ideal biocompatibility and bioactivity is considered as a perfect material for orthopedic applications because the direct contact between the underlying implant and bone tissue is inhibited and in turn, the release of challenging ions from the implant is decreased[55]. Further, high bonding strength may be provided with underlying substrate. The chemical stability and the low degradation rate in biological environment influences their long-term durability[6,34]. Also, the coating material with nanostructural configurations is favorable for the absorption of ions such as Ca^2+^ and magnesium (Mg^2+^)[56-59], which result in better osteoconductivity[60]. The other features that may influence the establishment of good bonding strength between the underlying implant and coating *in vitro* and *in vivo* include surface roughness, thickness, microstructures[6,35,61], Young’s modulus and CTE[62,63]. Rough surface is favorable for cell attachment and proliferation, which are valuable for bone implant fixation[64]. However, the presence of microcracks in the surface is not advantageous for corrosion resistance and the good bonding strength[65].

### Cell-coating interaction

**Fig. 1:**
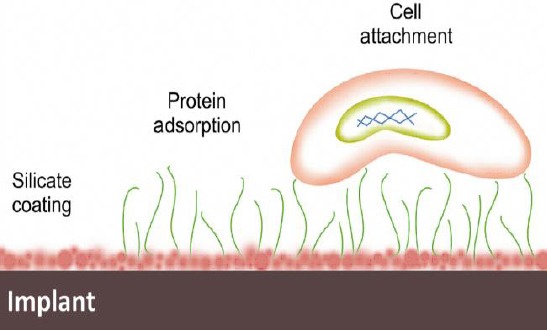
The effect of released ions on osseointegration and antibacterial properties.

Biological reactions are generally occurred on the surface; therefore, thesurface characteristics of coating such as ion release and topography are key factors in the implant-cell interactions[66-68] (Fig. 1).

**Fig. 2:**
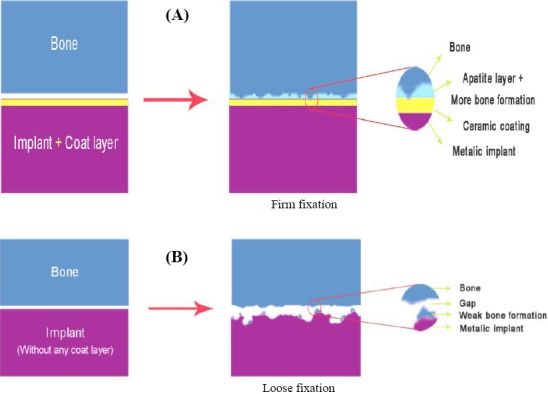
The effect of ceramic coating on the Ti substrate. (A) The implant without coating leads to weak bone formation and the loosening of the implant; (B) the apatite formation on the implant with coating resulted in more bone formation and tight fixation of implant.

As indicated in Figure 2, the surface properties of the implant are improved by coating, and apatite formation is induced on the surface leading to a better bonding with bone tissue (Fig. 2A) compared to uncoated substrate (Fig. 2B). The formation of a silica layer on the surface is beneficial to the adsorption of proteins. This silica layer supports and facilitates the interactions between proteins and the surface of material and, in turn, affects cell behaviors[69]. Hence, the cell-material interaction may be effective in establishing a tight bonding with the host bone tissue, which provides a suitable substrate for cell attachment. Also, it is notable that the cell proliferation rate is related to initial cell attachment density[63].

The surface chemistry may affect the adsorption of proteins from the surrounding medium to facilitate the cell attachment[70]. Also, more binding sites can be provided for the adsorption of protein by Si^4+^ ions[71]. Briefly, the molecular mechanism by which the interaction is established between the cells and underlying substrate may be described as follows.

After *in vitro* and/or *in vivo* implantation, several biological reactions occur on the surface of implant. First, proteins are immediately adsorbed to the surface of implant[72]. Next, integrins may be bound to proteins, which transduce extracellular signals inside the cells[68,69]. As a result of these signaling pathway, the cell behavior can be altered through the regulation of those genes whose functions are associated with attachment, proliferation and differentiation. Herein, the characteristics of the surface may determine the orientation of adsorbed proteins and the expression of integrins[70].

When the coated implant is placed *in vivo*, the coating materials are exposed to physicochemical and/or cell-mediated dissolution and corrosion. As a result, it can be degraded and replaced by newly formed bone tissue[73]. Therefore, it is suggested that the release of ions from the bioceramic coating controls the local microenvironment, which determines the host cell behavior and supports the new bone formation process. It is thought that the chemistry and the microstructure of the surface are responsible for advantageous stimulatory effect.

### Trace element-modified calcium silicate ceramic coating

The CaSiO_3_ and Ca_2_SiO_4_ coatings have shown to have excellent *in vitro* bioactivity. In addition, these types of coatings demonstrate a rough microstructure and higher bonding strength compared to HA[6,29,32,33]. Nonetheless, both HA and CaSiO_3_ coatings possess rapid degradation rate, which resulted in disintegration of the coatings and compromising their bonding strength and implant fixation[74]. Although there are no microcracks between the Ca_2_SiO_4_ coating and the substrate[29], the short-term osseo-integration[29,75,76] and poor chemical stability[49] are major problems that hinder the *in vivo* long-term durability of implants.

It is known that the incorporation of ions into CaO-SiO_2_ improves the chemical stability and mechanical properties compared to HA and CaSiO_3_. In addition, ion-modified CaO-SiO_2_ materials have apatite-forming ability in simulated body fluids[51,52].

The feedstock (CaO-ZrO_2_-SiO_2_ [CZS]) is one of the Zr-modified materials. The atmospheric plasma or air plasma (APS)-sprayed CZS on Ti-6Al-4V substrate[77] has exhibited a higher bonding strength than plasma-sprayed HA coating[22]. This higher bonding strength of CZS coating is attributed to the large content of zirconia in the CZS coating. Also, CZS coating has high strength and good toughness due to the comparable CTE of CZS coating and Ti-6Al-4V[78,79]. It has been shown that the *in vitro* cytocompatibility of CZS coating on Ti substrate can promote the adherence of a large number of canine marrow stem cells (MSCs) to the material[77]. Furthermore, the MSCs well proliferate on CZS, which can be due to the rough surface of coating. However, the cell proliferation rate of CZS and HA is similar. A report has demonstrated that bone marrow-derived stromal cells (BMSCs) firmly adhere to the surface of CZS coating and show a considerably faster cell proliferation compared to HA coating[79]. It has been suggested that the presences of Si^4+^ ions positively affect the cell behavior. In addition, silicon-enriched layer formed on the surface of CZS is beneficial to protein adsorption and cell attachment[79].

The second Zr-modified material is Baghdadite (Ca_3_ZrSi_2_O_9_). The Ca_3_ZrSi_2_O_9_ coating on the Ti-6Al-4V substrate using APS has been shown to have stronger bonding strength with Ti substrate[80] compared to plasma sprayed-HA coating[81]. Although the surface roughness of Ca_3_ZrSi_2_O_9_ is higher than CZS, it possesses lower bonding strength.

There are different Mg-modified compounds that show good bonding strength and better biocorrosion and antibacterial properties compared to HA and β-TCP. These compounds include akermanite (Ca_2_MgSi_2_O_7_), diopside (CaMgSi_2_O_6_), bredigite (Ca_7_MgSi_4_O_16_), merwinite (Ca_3_MgSi_2_O_8_) and monticellite (CaMgSiO_4_)[52].

The Ca_2_MgSi_2_O_7_-coated Ti-6Al-4V by APS[48] indicated that the bonding strength of the coating is much higher than HA[22,36,82]. However, the mismatch of CTE between Ca_2_MgSi_2_O_7_ and underlying Ti substrate leads to the formation of longitudinal cracks inside the coating. Thus, the bonding strength of Ca_2_MgSi_2_O_7_ is lower than CaMgSi_2_O_6_ due to the presence of microcracks.

The CaMgSi_2_O_6_-coated Ti-6Al-4V using plasma spray has exhibited higher bonding strength compared to HA[34]. This higher bonding strength is due to the comparable CTE of CaMgSi_2_O_6_ and underlying Ti substrate, which prevents the formation of microcracks at the interface[34].

**Fig. 3:**
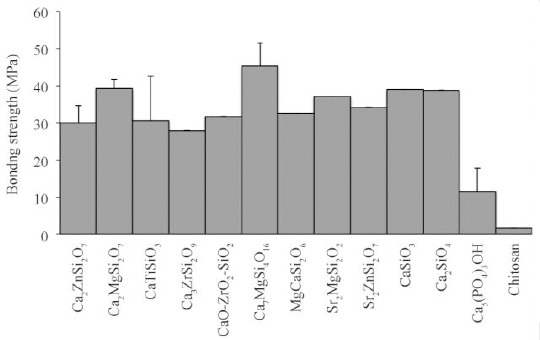
Bonding strength of coating reported in the literatures for hardystonite (Ca_2_ZnSi_2_O_7_)[49,93], akermanite (Ca_2_MgSi_2_O_7_)[48], sphene (CaTiSiO_5_)[20,35,93], baghdadite (Ca_3_ZrSi_2_O_9_)[80], CaO-ZrO_2_-SiO_2_ feedstock (CZS)[77], bredigite (Ca_7_MgSi_4_O_16_)[83], diopside (MgSi_2_O_6_)[34], Sr_2_MgSi_2_O_7_ (SMS)[42], Sr_2_ZnSi_2_O_7_ (SZS)[100], CaSiO_3_[6], Ca_2_SiO_4_[29], hydroxylapatite (Ca_5_(PO_4_)_3_OH)[6,22,29,32-34,36,49,81,82] and chitosan[13,111]. Also, for baghdaditeand akermanite, there was no accurate value for bonding strength; a range of bonding strength value was reported.

Ca_7_MgSi_4_O_16_ can also be applied as a coating material on the implant surface. When Ca_7_MgSi_4_O_16_ is coated on the Ti-6Al-4V surface[83], the bonding strength is higher than HA[22], wollastonite[84], Ca_2_SiO_4_[29], CaMgSi_2_O_6_[34], CaTiSiO_5_[35] and Ca_2_MgSi_2_O_7_ coatings[48]. This high bonding strength is mainly due to the tight interface between coating and underlying surface, no clear microcracks and well-melted Ca_7_MgSi_4_O_16_ powder. The BMSCs adhere well on the surface with a higher proliferation rate than HA. This is ascribed to the capability of bone-like apatite layer enhancing the osteoblastic activity[85-87] and stimulating the role of Mg^2+^ and Si^4+^ ions[88-91]. Although both Ca_2_MgSi_2_O_7_ and Ca_7_MgSi_4_O_16_ showed bonding strength higher than HA, Ca_2_MgSi_2_O_7_ had lower bonding strength compared to Ca_7_MgSi_4_O_16_ due to microcracks (Fig. 3).

Ca_3_MgSi_2_O_8_ and CaMgSiO_4_ are the next materials with a potential use as coating. The CTE of both is closer to that of Ti-6Al-4V alloy[92]. However, no data are available in the literature focusing on their applications as coating on Ti-6Al-4V substrate.

Ca_2_ZnSi_2_O_7_ is the other ion-modified material with enhanced mechanical, biological and antibacterial properties. The coating of Ca_2_ZnSi_2_O_7_ on Ti-6Al-4V surface through APS obtained the higher bonding strength compared HA coating[93] mainly because of their comparable CTE[94]. The plasma- sprayed Ca_2_ZnSi_2_O_7_ on Ti-6Al-4V surface also showed a significantly higher bonding strength than HA[49]. Further, the coating supported primary human osteoblasts cell and osteoblast-like cell line (MC3T3-E1) attachment, spreading and proliferation[49,55,95] due to the presence of Ca^2+^ and Si^4+^ ions[93]. Moreover, this coating demonstrated a higher bone interface contact and faster osseointegration compared to CaSiO_3_ without the formation of fibrous tissue. Besides the osteogenic properties, Ca_2_ZnSi_2_O_7_ is able to show antibacterial effect against *Escherichia coli* and *Staphylococcus aureus*[49,95]. This antibacterial activity is thought to be related to the initial damage to cell wall and cell membrane.

CaTiSiO_5_ is a Ti-modified material used as a coating due to the close CTE to Ti-6Al-4V[35,96]. The CaTiSiO_5_ coating on Ti-6Al-4V demonstrated a bonding strength considerably higher than HA and Ca_2_ZnSi_2_O_7_[93]. This superior bonding strength of the CaTiSiO_5_ compared to Ca_2_ZnSi_2_O_7_ is probably due to the presence of Ti^4+^ in the CaTiSiO_5_, which may improve the chemical and diffusion bonding between CaTiSiO_5_ and the underlying Ti-6Al-4V substrate[97]. However, the Ca_2_ZnSi_2_O_7_ showed a rougher surface compared to CaTiSiO_5_. It should be noted that CaTiSiO_5_ coating on Ti-6Al-4V can be prepared by sol-gel spinning[20]. The prepared CaTiSiO_5_ showed a higher bonding strength than HA but lower than plasma-sprayed CaTiSiO_5_. The higher bonding strength is thought to be related to the inherent properties of CaTiSiO_5_. However, both soaking the Ti-6Al-4V implant in CaTiSiO_5_ sol-gel solution and HA showed high bone-implant contact, while uncoated Ti-6Al-4V revealed a significant poor bone-implant contact due to the presence of wide fibrous tissues. Moreover, both HA and CaTiSiO_5_ coatings exhibited comparable mechanical fixation but CaTiSiO_5_ showed considerably higher mechanical fixation compared to the uncoated Ti-6Al-4V[96]. Nonetheless, CaTiSiO_5_ coating indicated higher bonding strength compared to sol-gel spinning but lower strength than plasma-sprayed coating.

The CaTiSiO_5_ coating on Ti-6Al-4V through plasma spray shows no microcracks at the interface and reveals a strong bonding strength[35] higher than HA[22,33,81,98]. Additionally, the CaTiSiO_5_ coating could support human osteoblast-like cell attachment, spreading and proliferation, which is due to the presence of Ca^2+^ and Si^4+^ ions. The Ca_2_ZnSi_2_O_7_ coating, however, demonstrates a higher proliferation rate than CaTiSiO_5_ and Ti-6Al-4V substrate, which is related to the release of Zn^2+^ ions from the Ca_2_ZnSi_2_O_7_[93].

Evidence has shown that different methods can be used for preparation of CaTiSiO_5_ coating. Each of the preparation methods has its own advantages and disadvantages. According to the previous reports, the plasma spray technique produces a much denser microstructure compared to the sol-gel method. Nonetheless, using sol-gel method, the coating could be sintered in low temperatures since at higher temperature, it will oxidize and damage the surface of underlying substrate. In addition, the problem of low temperature sintering is that a completely dense microstructure cannot be obtained as observed for sol-gel method, thus affecting the bonding strength[35]. However, the advantages of the plasma spray method as a frequently commercial method for the preparation of coating is high deposition rate and rough surface, which is favorable for bone substitute[6,21].

It is worth noting that the simultaneous incorporation of ions into Ca-Si system is also possible to further improve biological and mechanical integrity. Recently, Sr^2+^ and Ti^4+^ have incorporated into Ca-Si and improved the bioactivity and the proliferation of mesenchymal stem cell compared to Ca_2_ZnSi_2_O_7_[99]. This nanocomposite may have the potential to be used as a coating. An investigation has indicated that when Sr^2+^ and Zn^2+^ are incorporated into Ca-Si structure, Sr_2_ZnSi_2_O_7_ (SZS) is formed. The SZS considerably controlled the inflammation, decreased the osteoclastogenesis and improved osteogenesis with higher bonding strength compared to HA[100]. The reason is that both Sr^2+^ and Zn^2+^ are found in the structure of natural bone tissue and have stimulatory effect on bone formation. In addition, there were no microcracks at the interface mainly due to the similarity of CTE. Moreover, the presence of Zn^2+^ may induce anti-inflammatory effects after implantation.

Other study has reported that the incorporation of Sr^2+^ and Mg^2+^ into Ca-Si system results in the formation of Sr_2_MgSi_2_O_7_ (SMS). This modified coating represented higher capacity to prevent osteoclastogenesis with stronger bonding strength compared to HA. This property is due to the similarity of CTE of coating and substrate as well as the absence of microcracks on the surface of coating[42]. Also, this coating has higher bonding strength than SZS (Fig. 3).

**Fig. 4:**
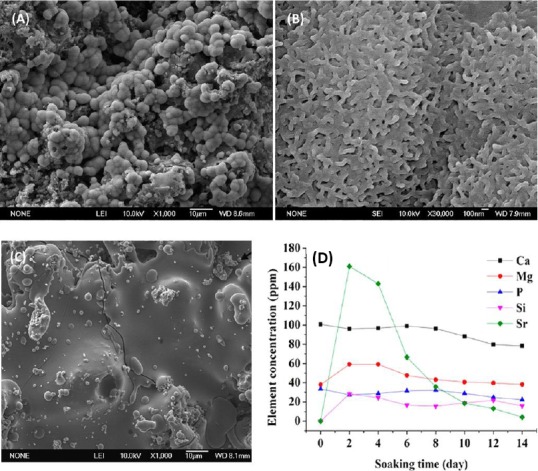
Scanning electron microscopy images of (A) the apatite layer formed on the surface of SMS coating after immersion in simulated body fluids, (B) lath-like morphology of apatite layer and (C) Ti alloy without coating, indicating that the SMS coating improves the bioactivity if Ti alloy. (D) Release of Sr^2+^ from SMS coating, which is considerably higher than that observed for HA coating, showing possible mechanism for reduced osteoclastogenesis of SMS coating. Reproduced with permission[42], Copyright 2014, ACS applied materials & interfaces.

**Fig. 5:**
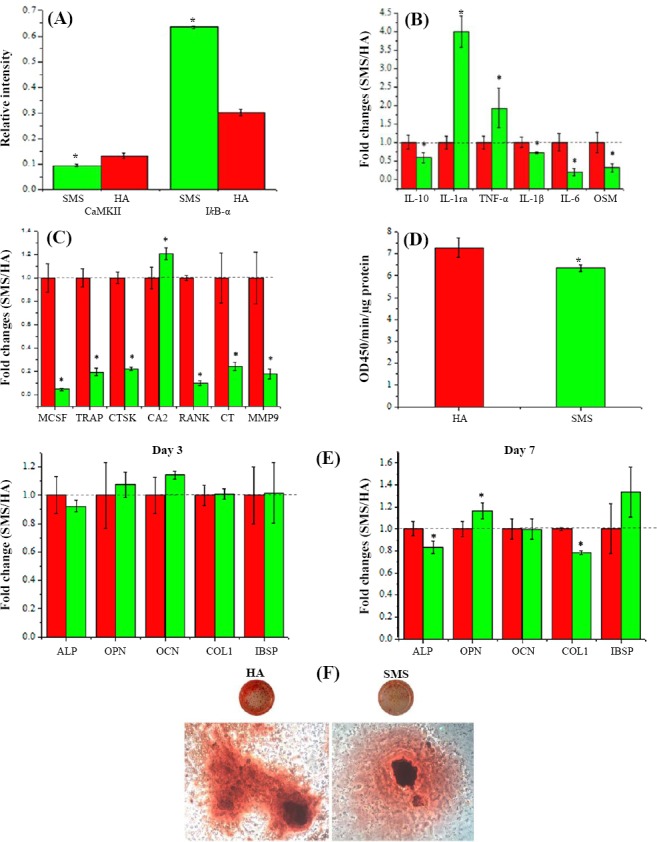
The mechanism by which SMS coating hinders the inflammatory response. A significant decrease in Ca^2+^ and an increase in Mg^2+^ and Sr^2+^ concentrations inhibit the formation of fibrous capsule by Wnt and Ca^2+^ pathway (Wnt/Ca^2+^)-related genes and toll-like receptors pathway. (A) Expression of calmodulin-dependent protein kinase II (CaMKII) and nuclear factor of kappa light polypeptide gene enhancer in B-cells inhibitor, alpha (I*k*B-α). (B) Chages of inflammation-related genes including interleukin 10 (IL-10), interleukin-1 receptor antagonist (IL-1ra), tumor necrosis factor alpha (TNF-α), interleukin-1 beta (IL-1β), interleukin 6 (IL-6) and oncostatin M (OSM). (C) the activities of osteoclastogenesis- and osteoclast-related genes, including macrophage-colony stimulating factor (MCSF), tartrate-resistant acid phosphatase (TRAP), cathepsin K (CTSK), carbonic anhydrase II (CA2), receptor activator of nuclear factor k B (RANK), calcitonin (CT) and matrix metalloproteinase-9 (MMP9). (D) ALP activities of HA and SMS coatings (E). Osteogenesis-related gene expression, including alkaline phosphatase (ALP), osteopontin (OPN), osteocalcin (OCN), collagen type I (COL1) and integrin-binding sialoprotein (IBSP) by BMSCs in days 3 and 7. (F) Bone mineralization of HA and SMS coatings. * shows significant difference (*P*<0.05). Reproduced with permission[42], Copyright 2014, ACS applied materials & interfaces.

As an example of the *in vitro* bioactivity of these modified ceramic coatings, after soaking SMS in simulated body fluids solution, a lath-like apatite is formed on the surface (Fig. 4). Unlike HA coating, the SMS coating is able to prevent inflammatory reaction. The mechanism by which SMS coating inhibits the inflammatory response is that a significant decrease in Ca^2+^ and an increase in Mg^2+^ and Sr^2+^ concentration are occurred, and the formation of fibrous capsule is inhibited by Wnt/Ca^2+^ pathway after implantation[101]. Also, Mg^2+^ and Sr^2+^ can decrease inflammatory cytokine production[102,103]. Mg^2+^ is known to suppress inflammatory cytokine production via the inhibition of toll-like receptors pathway[104] (Fig. 5). However, the mechanism for inhibitory effect of Sr^2+^ is not fully understood. It may be speculated that the possible mechanism for reduced osteoclastogenesis of SMS coating is due to released Sr^2+^ from the coating (Fig. 4)[105]. However, the osteogenic differentiation of BMSCs on SMS is similar to HA. This fact reveals the similar *in vitro* osteogenic-inducing capability of SMS and HA.

EPD accompanied with micro arc oxidation (MAO) is another known method for coating of modified Ca-Si ceramic coating on the metallic substrate[106]. The advantages of the EPD method include the possibility of using versatile materials, cost-effectiveness, application of simple equipment, storage at room temperature, coating in a short time and less restriction applied to substrate shape[107]. In particular, the EPD method is able to produce uniform coating on the substrate compared to other coating techniques. In addition, it has been found that MAO layer is porous with high adhesion strength[108]. Furthermore, MAO is recognized as an effective approach to control the corrosion rate of biodegradable Mg alloy. Therefore, both corrosion resistance and bioactivity of substrate could be enhanced[109,110]. Best of our knowledge, this method has not been used for preparation of modified Ca-Si ceramic coating on Ti substrate. Thus, the preparation of modified Ca-Si ceramic on the Ti substrate using EPD could be the topic of studies in the future. Moreover, the biological response at the tissue-implant interface of surface-modified metallic implants and their *in vivo* mechanism must be carefully identified for new applications and enhance the functionalities of the future generations of medical implants.

In summary, all modified Ca-Si ceramic coatings show higher bonding strength compared to HA and polymeric coating such as chitosan. This high bonding strength can be mostly due to the comparable CTE between coating and substrate, their microstructure and preparation method. This silicate coating improves degradation rate and forms an apatite layer on the surface. In addition, the higher bonding strength of these coating materials is valuable for *in vivo* implant tissue integration, indicating that the stress at the implant-tissue interface is decreased, and biological stability and lifetime of the implant are improved.

This review discussed that the methods of coating preparation would lead to different bonding strength values. For example, the CaTiSiO_5_ prepared by sol-gel spinning has shown to have a bonding strength considerably lower than that of prepared by plasma spray method. This issue indicates that different preparation methods may have influence on the properties and the performance of coatings. Also, there are few *in vivo* studies focusing on these modified coating Ti-6Al-4V substrate. In addition, post-real time evaluations such as magnetic resonance imaging are useful for better understanding of their biological performance.
